# Heterogeneity among Homeless Australian Women and Their Reasons for Homelessness Entry

**DOI:** 10.3390/ijerph19158909

**Published:** 2022-07-22

**Authors:** Wayne A. Warburton, Marina Papic, Elizabeth Whittaker

**Affiliations:** 1 School of Psychological Sciences, Macquarie University, Sydney, NSW 2109, Australia; 2 School of Education, Australian Catholic University, North Sydney, Sydney, NSW 2060, Australia; marina.papic@acu.edu.au; 3 Centre for Epidemiology and Evidence, NSW Ministry of Health, St Leonards, NSW 2065, Australia; elizabeth.whittaker@health.nsw.gov.au

**Keywords:** homeless mothers with children, homeless lone women, specialist homelessness services, homelessness service delivery

## Abstract

Many women become homeless each year, both women who are alone and women with children. Both groups face substantial risks to their physical and mental health, as do the children of homeless mothers. Little is known about the similarities and differences between these two groups in terms of their demographic characteristics, their circumstances on presentation to specialist homelessness services, and the factors that have contributed to their homelessness. The current study analysed data from 163 single mothers with children and 126 lone women who presented to a specialist homelessness service in Australia. It found some similarities between groups, but also considerable heterogeneity. Single mothers were more likely to be younger, to have been born overseas, and to have been homeless in the past 12 months. Lone women were more likely to have medical issues, a mental health condition, addiction issues, admission to a psychiatric ward in the past 12 months, and to not be in the labour force. Implications for service delivery are discussed.

## 1. Introduction

Family homelessness continues to be a prominent concern in both urban [1,2,3,4] and rural [5] Australia, with reports suggesting that although the numbers of homeless families are underestimated due to being ‘invisible’ to some services [5,6], they are arguably the fastest-growing subpopulation of homeless Australians [5,7,8]. Of particular concern is the growing number of homeless mothers with children due to their vulnerability. In Australia, McAuley Community Services for Women wrote in their 2020 submission to a government homelessness inquiry that ‘over the past decade the profile of a person who is homeless has changed: that person is now, typically, a woman who has experienced family violence, and is the sole parent of at least one child’ ([9], p. 3). Indeed, family and domestic violence (FDV) is the key reason women and children leave their homes in Australia. Women experiencing FDV make up 42% of the clients of services that specialize in assisting homeless Australians (i.e., specialist homeless services (SHSs)) [10]. Of the 278,300 Australians who accessed SHSs in 2020–2021, ~117,300 were families, ~116,200 were members of families who had experienced domestic or family violence, and the largest single group was lone parents with one or more child (~84,900) [10]. Around 39% of women (>52,000) were single mothers with one or more children [10].

It is not just single homeless mothers who are at significant risk within the homeless population. The European Federation of National Organisations Working with the Homeless (FEANTSA) note that ‘homelessness services have been traditionally designed for male users, ignoring the specific needs of homeless women as well as their different pathways into and out of homelessness’, a situation that brings concomitant health and service delivery implications for both homeless lone women and homeless women with children [11]. For example, many homeless women have complex health issues, including those arising from trauma and abuse [11]; homeless women are more likely to rely on precarious and dangerous supports to survive [12]; and homeless women often face unique challenges, such as accessing menstruation products and maternal health care and gender inequality in employment [13].

Throughout Australia, homelessness responses for Australian women, particularly women fleeing FDV, are largely crisis based, where short-term accommodation options, such as rooms in shared facilities or apartments or even motel accommodation, are provided [14]. Unfortunately, the supply of SHSs does not accommodate the current demand, with 32.3% of Australians seeking SHSs in 2020–2021 having their need unmet, including 48.2% of people seeking help in the largest state, New South Wales [15]. Over two-thirds of unassisted requests for SHSs are from women [10], although it is hard to extrapolate from data the proportions that are lone women and mothers with children. These figures are concerning because many homeless women are escaping abuse and violence [10,16], and women who attempt to escape violence and abuse are at greater risk of being injured or seriously hurt, or even losing their life due to violence by their partner [17]. Thus, an inability to offer emergency accommodation may mean that such women are forced to return home to an unsafe environment. In addition, the health and wellbeing of homeless women is precarious [11], with the health of homeless mothers also inextricably linked to that of their children [18,19], making access to SHSs for these women and children crucial.

Homelessness, combined with exposure to other challenges their parents are facing, makes homeless children particularly vulnerable to a broad range of complex and inter-related social, educational, and health risks and associated needs [18,20]. Children in homeless families are more exposed to stress and have fewer resources than low-income children of similar backgrounds whose families are housed [21]. The National Center on Family Homelessness in the US reported that children who have experienced homelessness are often denied stimulating early play and educational experiences that develop the neural pathways in the brain that are foundational for academic readiness, positive social skills, and emotional stability [22]. Consequently, children of homeless parents are more likely to experience poor physical and emotional health, developmental delays, behavioural problems, poor literacy and numeracy, and poor educational outcomes [22,23,24,25]. Homeless mothers have significantly higher psychological stress and distress, often have less than optimal parenting practices, and have higher rates of depression. Their children are consequently more likely to experience lower self-worth and greater behavioural and emotional problems [26,27,28]. Of notable concern is that child homelessness is predictive of homelessness as an adult [29]. For example, Flatau and colleagues [30] found that nearly half of the 647 surveyed clients of homelessness services (49%) reported that their parents had also been homeless at some point. Of the total, 284 were women, 167 were single women, 77 were families, and 106 were women or women with children escaping domestic violence, suggesting that intergenerational homelessness is likely an issue in all these populations.

Even though homelessness among lone women [31] and single mothers and their children [11,16] is becoming more widespread, with negative impacts on affected women and children, little research has been conducted in Australia on either group, and there is very little international research that has compared the experiences of homelessness for both groups. In a 1989 US study, Burt and Cohen [32] found that these groups did not differ much in terms of length of homelessness and joblessness, poor health status, and the percentage of those who do not get enough to eat. However, they also found distinct differences between homeless mothers with children and homeless lone women. Lone women were more likely to be chronically homeless, older, substance dependent, and diagnosed with a mental health condition. Mothers with children tended to have more poverty indicators—lower income, less employment, and greater reliance on food stamps to eat. In a further 1993 US study, North and Smith [33] compared homeless lone women, lone men, and mothers with children. They found that compared with homeless mothers with children, homeless lone women were more often Caucasian, were more often employed, had a longer history of homelessness, and were more likely to have a history of alcoholism or schizophrenia. North and Smith concluded that the population of homeless women is heterogeneous with at least two subgroups (lone women vs. mothers with children). The differences between these groups likely contribute to different pathways of entry into homelessness, with implications for one-size-fits-all programs.

Apart from the factors noted by Burt and Cohen [32] and North and Smith [33], there are a number of other factors linked to homelessness entry that are potentially relevant to homeless lone women and/or homeless women with children: housing crisis, housing affordability stress, inadequate and/or inappropriate housing, financial difficulties, FDV, low levels of support from family and community, relationship breakdown/issues, transition from custodial arrangements or care, nondomestic violence or abuse, discrimination, and not being able to return home due to the home environment [34]. These factors may also be important points of difference between these two groups. Indeed, given the age of existing papers that provide relevant comparisons, it seems timely to examine points of difference between the two groups across all factors.

In addition, given that the Australian government’s 2021 inquiry into homelessness specifically encouraged ‘data collection that enables the experiences and needs of [homeless] children and their families to be identified, so that it can inform policy planning and service delivery’ [35], and there are growing concerns in Australia about lone homeless women [31], research on both groups in the Australian context seems both warranted and timely. It seems especially important to understand the ways in which these two groups differ on the factors noted earlier so that services can better provide diversified services that can meet the needs of both. The current study aims to address these knowledge gaps by ascertaining and comparing the various ways that lone women and single mothers and their children become homeless. Once identified, the factors that contribute to mothers and their children becoming homeless can inform future policy formulation intended to reduce family homelessness.

## 2. Materials and Methods

### 2.1. Design

The authors were granted clearance to access the program database of a homelessness support agency in Melbourne, Victoria, Australia. The de-identified dataset comprised all clients who presented to the support agency over a 22-month period (January 2013 to October 2014), which enabled cross-sectional analyses to be conducted. The use of these historical data is justified by (a) the service losing funding soon after, with newer data not able to be obtained, and (b) the demographic makeup of homelessness in Australia remaining similar in the intervening time, in terms of the percentage of homeless lone parents, homeless women, and homeless mothers with children. For example, the percentage of lone parents using SHSs was 31% in 2011–2012, 31% in 2012–2013, 33% in 2013–2014, and 33% in 2020–2021 [10,36], and the percentage of women using SHSs was 59% in 2013–2014 and 60% in 2020–2021 [10,36]. Further, the (Australian) Council of Single Mothers and their Children, in their 2018 national survey, found that one in eight single mothers faced insecure housing, and referencing their 2007 study, concluded that ‘the housing situation faced by many single mother families today has been dire for over a decade’ ([37], p. 33).

All clients had given prior written consent for their data to be used for research purposes at the time of their first presentation. No records were omitted due to incomplete data.

### 2.2. Sampling

A total of 779 clients presented to the support agency during the timeframe. Clients were retained in the dataset if they were female (N = 442), presented to the service as the head of the family (N = 381), and reported their living arrangement at the time of presentation as either ‘lone person’ or ‘one parent with child(ren)’ (N = 302). From these 302 cases, individuals who presented more than once to the service during the timeframe had their first presentation retained and data from subsequent presentations removed (N = 13), as the aim of the study was to determine why women presented to homelessness support agencies in the first instance. The final database contained data from 289 participants.

### 2.3. Measures

#### 2.3.1. Sociodemographic Characteristics

Participants provided data about basic demographic information, including age, ethnic background, homelessness status, and mental health history.

#### 2.3.2. Circumstances upon Presentation

Upon presentation to the support agency, participants were asked to indicate whether they were currently dwelling in their own home or if they were homeless (primary homelessness: sleeping rough on the streets or sleeping in a car; or secondary homelessness: boarding house/hostel, shelter/refuge, caravan park, or couch surfing). Participants also reported their employment status and main source of income at the time of presentation.

#### 2.3.3. Reasons for Presentation

Participants were asked to report their primary reason for presenting to the support agency, as well as any other factors that may have contributed. They were provided with a list of possible factors derived from the Specialist homelessness services collection manual-July 2013 [34]. These included housing crisis, previous accommodation ended, relationship breakdown and fleeing domestic violence (full list of presenting reasons in Results).

#### 2.3.4. Analysis Strategy

Sociodemographic characteristics were compared by homeless group (lone women versus single mothers with child/ren). Results were summarised using descriptive measures expressed as mean (standard deviation (SD)) for continuous variables and number (percent) for categorical variables. Measures of central tendency and variance were checked for continuous variables before adopting mean (SD) for summary statistics. Unadjusted differences between groups were tested using a chi-squared test for categorical variables and the *t*-test for continuous variables. Statistical significance was set at the *p* < 0.05 level. All analyses were conducted using IBM SPSS software.

## 3. Results

### 3.1. Sample Characteristics

Of the 289 women who presented to the support agency, 56.4% were homeless single mothers with child/ren (N = 163), and the remaining 43.6% were lone women (N = 126). On average, homeless single mothers had 1.71 (SD = 1.16) children in their care.

As shown in Table 1, there were a number of significant differences between the two groups. Single mothers with child/ren were younger than lone women and were less likely to have been born in Australia or to speak English at home. Single mothers with child/ren were also more likely to have experienced homelessness in the 12 months before presentation compared with lone women. Lone women were significantly more likely than single mothers to have been diagnosed previously with a mental health condition, and a greater proportion had been admitted to a psychiatric hospital or unit in the 12 months before presentation. No differences between the groups were found for Aboriginal or Torres Strait Islander descent or admission to a hospital for nonpsychiatric reasons in the past 12 months.

### 3.2. Circumstances upon Presentation

Table 2 shows the proportions of women experiencing different circumstances in relation to dwelling type, labour force status, and main source of income upon presentation to the support agency. As to dwelling type, a larger proportion of single mothers with child/ren reported secondary homelessness; that is, they were living in transitional accommodation (e.g., boarding house/hostel, shelter/refuge, caravan park, sleeping in a car, or couch surfing) at the time of presentation. Almost no women in either group reported primary homelessness.

Although levels of full employment were low, a larger proportion of single mothers with child/ren than of lone women reported being currently unemployed, whereas a majority of lone women reported that they were not currently seeking employment. In keeping with these trends, the vast majority of lone women reported government benefit or allowance as their main source of income, and most single mothers reported a government benefit or allowance or parenting payment as their main source of income.

### 3.3. Contributing Factors toward Presentation to a Homelessness Service

On presentation to the support agency, participants were asked to identify the main reason for their presentation to the homelessness service and to indicate other contributing factors (Table 3). The main reason reported by the largest proportion of both groups was housing crisis, followed by financial difficulties and inadequate or inappropriate dwelling conditions. A significantly greater proportion of lone women identified mental health issues or problematic addictive behaviours as the main reason for their needing to access a homelessness service.

More than one quarter of both groups reported inadequate or inappropriate dwelling conditions, housing affordability stress, or lack of family/community support. For lone compared with single mothers, mental health issues, problematic addictive behaviours, and medical health issues were significantly more common contributing factors for seeking a homelessness service. Conversely, a greater proportion of single mothers with children than lone women reported their previous accommodation ending as a contributing factor. For around 3 in 10 single mothers with children, domestic/family violence was the key factor or a contributing factor to their homelessness.

Correlation analyses were conducted to ascertain the predictors of homelessness that most strongly discriminated between homeless lone women and homeless mothers with children. Being a homeless mother with children was correlated with previous accommodation ending (*r* = 0.16, *p* = 0.008). Being a homeless lone woman was correlated with presenting to a SHS because of substance use (*r* = −0.31, *p* < 0.001), alcohol use issues (*r* = −0.23, *p* < 0.001), mental health issues (*r* = −0.24, *p* < 0.001), medical issues (*r* = −0.18, *p* = 0.002), or being itinerant (*r* = −0.12, *p* = 0.049), as well as with not being homeless in the last month (*r* = −0.20, <001) and greater time since last having a permanent address (*r* = −0.14, *p* = 0.024).

Significantly correlated variables were entered into a regression analysis. Backward elimination was then used to produce the most parsimonious model. Homeless mothers with children were more likely than lone women to have been homeless in the past month in short-term or emergency accommodation due to a lack of other options (β = 0.22, *p* < 0.001). In contrast, lone women were more likely to have presented to the SHSs because of problematic drug or substance use (β = −0.20, *p* < 0.001), mental health issues (β = −0.16, *p* = 0.004), and alcohol use (β = −0.15, *p* = 0.008).

## 4. Discussion

As expected, there were some issues common to both groups. For example, as with homelessness generally, issues around housing affordability, housing suitability, inadequate housing, and financial issues were widely reported by both lone women and single mothers with children. These findings are not surprising. Homeless women are often fleeing domestic violence or abuse and are forced into homelessness with few assets and few housing options [38]. In addition, the rising cost of housing in many countries, including steep recent increases in Australian rental costs [39], and housing shortages in some areas of Australia [40] are forcing increasing numbers of people, including women, into homelessness. Johnson [41] notes that the key need for people who need to flee their homes (most often women) is emergency crisis accommodation. Unfortunately, in Australia, more than half of women seeking crisis accommodation are turned away due to lack of service availability [42].

This study also highlighted clear differences between lone women and single mothers with children who presented to a homelessness support agency in Australia. Two key findings emerged that support the notion that homeless women are a heterogeneous group. First, sociodemographic characteristics varied distinctly between lone women and single mothers. Second, while both client groups shared some reasons for presenting to the support agency, variances in their reasons for doing so may be due to the differences in the observed characteristics of the two groups. Most notably, homeless lone women were more likely to present to SHSs due to substance and mental health issues, whereas homeless mothers with children were more likely to seek SHSs because they had run out of other accommodation options.

In terms of demographic differences, single mothers with children were more likely to be younger (average age: 38 vs. 47), to have been born overseas, and to have experienced homelessness in the 12 months before presentation. The age difference is consistent with that found by Burt and Cohen [32], and the findings more widely accord with international research, which has revealed distinct differences between homeless families and other groups of homeless people [43]. Thus, the findings provide further support for the notion that in terms of homeless populations, tailored support and interventions may be a key approach [44]. For example, research from the US Institute of Children and Poverty [45] identified that teenage homeless mothers, compared with older homeless mothers, faced higher levels of discrimination and stigmatisation, were more likely to have experienced childhood abuse, had poorer relationships with their parents, were less knowledgeable about contraception, and were more likely to be sexually active at a younger age. These findings suggest that age differences exist among homeless mothers in relation to their sociodemographic characteristics and in relation to the issues they face during homelessness episodes. Consequently, even within a demographic such as homeless mothers, service provision may need to be tailored according to age profile. Additionally, there may be other subgroup differences amongst homeless mothers that are yet to be explored, including whether Indigenous or culturally diverse mothers require specific types of assistance to exit homelessness. Efforts to investigate preventative measures may also be valuable for subgroups, such as women who have migrated to Australia to reduce their likelihood of needing SHS support.

The analysis of sociodemographic differences also revealed that lone women were more likely to have a mental health condition and to have been admitted to a psychiatric hospital or unit in the 12 months before presentation, findings that align with those of Burt and Cohen [32] and North and Smith [33]. It is unclear why homeless lone women have been found to have higher rates of mental illness than their counterparts with children in three studies now, although it is feasible that this may partially reflect that some homeless women have had their children removed by social services due to mental illness [46]. It should also be noted that more than half the homeless mothers with children reported a mental health diagnosis as well, a rate itself noticeably higher than the general population, where 2014–2015 Australian Bureau of Statistics (ABS) figures show that 18% of Australians had a mental health or behavioural condition [47] and 2021 ABS figures show that 17% of Australians sought assistance for mental health [48]. Although these mental health issues may be caused or exacerbated by the stress and trauma the majority of these women have faced [49,50], it should be taken into account in any decisions about support for them, as homeless women with mental health issues require particularly flexible and intensive services [51]. Although some mental health services are available for homeless women in Australia, caps on federally funded assistance may be adverse for homeless women with extended or chronic mental health issues [9].

The data revealed that while almost all women had some type of accommodation, they were seeking the help of the homelessness service presumably because they either (1) felt at risk of becoming unhoused or (2) had been homeless in the past and were pre-empting entry into another homelessness episode. Nevertheless, clients gave clear reasons for presentation to the service, and it is noteworthy that while the two client groups reported a number of similar primary reasons for presenting to the support agency (housing crisis, financial difficulties, and inadequate or inappropriate dwelling conditions), there was also heterogeneity in both primary and contributing factors to their current or impending homelessness. These included differences in the levels of mental and health issues, physical health issues, problematic addictive behaviours, and previous accommodation ending with no alternative available. Interestingly, a higher proportion of lone women were not in the labour force, and a higher proportion of single mothers were in the labour force but currently unemployed, both factors that may have hindered these groups from finding and maintaining affordable housing. However, in terms of service provision, the differing underlying causes for not being employed suggest different supports needed for each group: one more tailored to gaining the skills to join the workforce, the other tailored to helping regain confidence and opportunities for employment. Together these findings suggest that women who are homeless are heterogeneous and require supports tailored to their differing needs.

A key way to respond to these diverse needs for Australian homeless lone women and mothers with children is a national homelessness strategy that recognizes their diverse needs, and structures government-funded services accordingly. The 2008 Australian national homelessness strategy had ambitious goals, but these have not been realised. In 2021, the Australian Parliamentary Standing Committee on Social Policy and Legal Affairs [52] recommended that a 10-year strategy needed to be developed, but this is yet to be actioned. Such a strategy could involve increasing crisis accommodation so that women fleeing FDV are no longer turned away from services, removing caps on the provision of mental health services to homeless women, and leveraging existing employment services so that specific assistance could be given to women who need to gain skills for employment versus those needing to regain employment confidence or find employment opportunities—that is, employment services could specifically target the diverse employment needs of both groups. Similarly, a national strategy could also involve extending existing financial counselling services to specifically provide bespoke assistance with the diverse financial issues faced by both groups of homeless women. Financial counselling services are free in Australia and are able to assist low-income clients in a range of ways: working with the client to maximise their income, including identifying all relevant available benefits; advocating on behalf of clients with creditors to waive or reduce debts or arrange affordable repayment plans; arranging vouchers to assist with the cost of utilities; providing referrals to free food provision and legal and employment services; and assisting with all aspects of managing on a very low income.

This study, which was focused specifically on the characteristics of single mothers with children facing homelessness, has provided an important initial contribution to the development of a sound Australian evidence base for policy to assist single mothers and their children, and lone women, in exiting homelessness. However, we acknowledge the study’s limitations. First, as an initial study limited in scope, it used historical data to analyse the program dataset of one metropolitan support agency. Further research is needed to establish whether the specific characteristics of lone women or single mothers differ between metropolitan or rural areas or across jurisdictions. Second, the cross-sectional design limits causal inferences about group membership and sociodemographic characteristics and the factors that contribute to homelessness entry. Third, the data collected relied exclusively on self-reporting, which is susceptible to memory error, nondisclosure, social desirability, or intentional misrepresentation. It should be noted, though, that past research (including our own [1]) has shown homeless people to be generally open and honest about their issues and concerns, and that self-reporting is the means by which this and all other reporting agencies obtain client information.

Despite these limitations, this study offers new insights into the heterogeneous homelessness pathways taken by these vulnerable groups, has implications for the nuanced delivery of services to these women (and children), and suggests several areas for future research. First, the efficacy of long-term supported housing options for lone women and for mothers and their children needs to be assessed, with the needs of both groups examined separately. While a body of literature exists on housing options for single chronically homeless men [53,54], it would be valuable to compare housing models that provide different levels of attached support to see the impact this has upon lone women and mothers and their children in the long-term. A second avenue for future research would be to examine in detail whether current programs address the differing needs of these two groups, and if not, what services and modes of delivery are needed to meet these needs.

## 5. Conclusions

Homeless women are a unique group. Within it, subgroups of lone women and single mothers with children can be distinguished by their sociodemographic characteristics and varied pathways into homelessness entry. This study has been a first step towards understanding how these groups can be identified and targeted for prevention and intervention efforts. It has shown that entry into homelessness for both lone women and single mothers often results from a complex interplay between a number of issues. Specialist homelessness services, which in Australia are currently stressed and under-resourced, need to be able to offer women the choice to stay in a safe place, rather than facing the undesirable situation of returning to an unsafe home or finding makeshift accommodation. However, given the heterogeneity found amongst homeless women in this study, funding to determine what SHS offerings are appropriate to cater to the differing needs of homeless lone women and homeless mothers with children seems warranted in order to best determine the way that the finite resources available can best achieve desired outcomes for them. Greater resource allocation to specialist homelessness services is also needed to enable tailored support to be provided to these women, particularly in the areas of mental health care and domestic or family violence.

## Figures and Tables

**Table 1:** Sociodemographic characteristics according to client group (N = 289).

Characteristics	Group				Group Differences
	Lone Women (N = 126)		Single Mothers with Child/ren (N = 163)		
Age (years): M (SD, range)	N = 126	47.22 (14.9, 22–90)	N = 163	38.33 (9.42, 19–66)	*t* = 6.18 (95% CI 6.06, 11.73) ***
Australian-born: N (%)	N = 126	96 (76.19)	N = 161	102 (63.35)	χ2 = 5.44 *
Speak English at home: N (%)	N = 102	96 (94.12)	N = 118	87 (73.73)	χ2 = 16.26 ***
Aboriginal or Torres Strait Islander descent: N (%)	N = 120	12 (10.00)	N = 158	11 (6.96)	χ2 = 0.83
Prior mental health diagnosis: N (%)	N = 87	69 (79.31)	N = 93	54 (58.06)	χ2 = 9.38 **
Prior history of homeless ^a^: N (%)	N = 121	28 (23.14)	N = 157	58 (36.94)	χ2 = 6.09 *
Hospital (excluding psychiatric reasons) ^a^: N (%)	N = 92	19 (20.65)	N = 91	14 (15.38)	χ2 = 0.86
Psychiatric hospital/unit ^a^: N (%)	N = 92	14 (15.22)	N = 91	0 (0)	χ2 = 15.00 ***

^a^ In the 12 months prior to presentation: * *p* < 0.05; ** *p* < 0.01; *** *p* < 0.001.

**Table 2:** Circumstances when presenting to support agency according to client group (N = 288).

Circumstances upon Presentation	Group	
	Lone Women (N = 126)	Single Mothers with Child/ren (N = 163)
Dwelling type: N (%)	N = 126	N = 162
Housed	117 (92.86)	125 (77.16) **
Homeless (primary)	2 (1.59)	0 (0)
Homeless (secondary)	7 (5.56)	37 (22.84) **
Labour force status: N (%)	N = 118	N = 150
Employed	3 (2.54)	8 (5.33)
Unemployed	36 (30.51)	71 (47.33) *
Not in the labour force	79 (66.95)	71 (47.33) **
Main source of income: N (%)	N = 122	N = 157
Wage/salary	2 (1.64)	5 (3.18)
Government benefit/allowance	116 (95.08)	73 (46.50) **
Parenting payment	2 (1.64)	76 (48.41) **
Other source of income	1 (0.82)	1 (0.64)

* *p* < 0.01; ** *p* < 0.001.

**Table 3:** Between-group comparisons of main reasons and other contributing factors that led to presentation at support agency according to client group (N = 287).

Presenting Reasons	Group			
	Lone Women (N = 125)		Single Mothers with Child/ren (N = 162)	
	Main Reason	Contributing Factor	Main Reason	Contributing Factor
Housing crisis ^	38 (30.40)	53 (42.40)	39 (24.07)	67 (41.36)
Financial difficulties	22 (17.60)	55 (44.00)	38 (23.46)	86 (53.09)
Inadequate/inappropriate dwelling conditions	18 (14.40)	39 (31.20)	25 (15.43)	46 (28.40)
Housing affordability stress	8 (6.40)	31 (24.80)	12 (7.41)	33 (20.37)
Domestic/family violence	4 (3.20)	20 (16.00)	15 (9.26)	36 (22.22)
Lack of family/community support	8 (6.40)	33 (26.40)	10 (6.17)	43 (26.54)
Mental health issues	10 (8.00)	53 (42.40)	3 (1.85) *	33 (20.37) ***
Problematic addictive behaviours ^a^	8 (6.40)	41 (32.80)	0 (0.00) **	6 (3.70) ***
Previous accommodation ended	0 (0.00)	4 (3.20)	5 (3.09)	19 (11.73) **
Relationship/family breakdown/time-out ^b^	2 (1.60)	28 (22.40)	3 (1.85)	40 (24.69)
Medical health issues	3 (2.40)	26 (20.80)	0 (0.00)	13 (8.02) **
Nonfamily violence	0 (0.00)	6 (4.80)	1 (0.62)	6 (3.70)
Unemployment or employment difficulties ^c^	1 (0.80)	20 (16.00)	0 (0.00)	12 (7.41)
Sexual abuse	0 (0.00)	5 (4.00)	0 (0.00)	4 (2.47)
Itinerant	0 (0.00)	5 (4.00)	0 (0.00)	1 (0.62)
Transition from custodial arrangements/foster care/other care arrangements ^d^	0 (0.00)	3 (2.40)	0 (0.00)	0 (0.00)
Discrimination	0 (0.00)	1 (0.80)	0 (0.00)	0 (0.00)
Unable to return home due to environmental reasons	1 (0.80)	1 (0.80)	0 (0.00)	0 (0.00)

^a^ Due to small numbers, ‘problematic drug use’, ‘problematic alcohol use’, and ‘problematic gambling’ were collapsed. ^b^ Due to small numbers, ‘time-out from family/other situation’ and ‘relationship/family breakdown’ were collapsed. ^c^ Due to small numbers, ‘unemployment’ and ‘employment difficulties’ were collapsed. ^d^ Due to small numbers, ‘transition from custodial arrangements’, ‘transition from foster care and child safety residential placements’, and ‘transition from other care arrangements’ were collapsed. ^ All statistics are in the format N (%) * *p* < 0.05; ** p < 0.01; *** p < 0.001. Note: ‘Disengagement with school or other education and training’ was removed as no participants reported this as a main or contributing factor.

## Data Availability

Data are available on request to the authors.
